# Generation of high-value products by photosynthetic microorganisms: from sunlight to biofuels

**DOI:** 10.1007/s11120-015-0182-1

**Published:** 2015-08-12

**Authors:** Alexandra Dubini, Taras K. Antal

**Affiliations:** National Renewable Energy Laboratory, 15013 Denver West Parkway, Golden, CO 80401 USA; Faculty of Biology, Moscow State University, Vorobyevi Gory, Moscow, 119992 Russia

Oxygenic photosynthesis is the singular important chemical process providing the energy source for almost all life on earth. It harnesses and stores sun energy in forms of high-energy intermediates, such as low potential electrons and ATP, used as energy sources primarily for the fixation of carbon from atmosphere into carbohydrates. The latter compounds supply carbon and energy to multiple anabolic processes associated with cell growth and division. In the past, photosynthesis provided significant energy storage in different forms of fossil fuels employed currently to satisfy human needs. With the increasing human population and expanding economy, there is a constant increase in consumption of limited fossil fuel reserves accompanied by the growing greenhouse gas emissions. Alternatively, photosynthesis has been suggested to have a great potential in providing ecologically safe sustainable production of renewable fuels from unlimited sources, viz. sun, water, and carbon dioxide. In fact global photosynthesis annually stores in biomass circa 10 fold more energy than can be used to power industry. Consideration of photosynthesis as a system for large scale fuel production would require elaboration of a set of biotechnological approaches to divert the flow of photosynthetic energy for synthesis of the desired chemical compounds instead of the normally produced carbohydrates. Oxygenic phototrophic microorganisms, such as green microalgae and cyanobacteria, are attracting considerable interest within these efforts due to their high photosynthetic conversion efficiencies, diverse metabolic capabilities, modest cultivation requirements, and ability to produce variety of useful compounds, including molecular hydrogen, oils, alcohols, hydrocarbons, valuable carotenoids, etc. without going through an intermediary biomass stage. However, specific problems associated with sustainable high-yield generation of these products remain to be addressed.

**Fig. 1:**
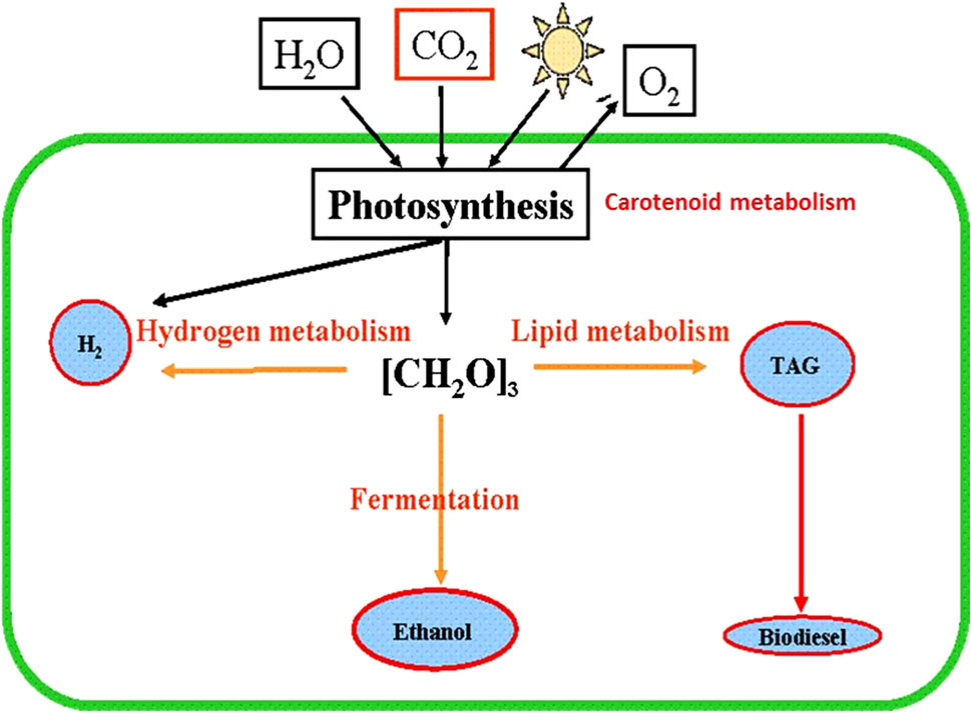
Metabolic pathways linked to biofuel production in green algae

This special issue provides an updated overview of the current state of knowledge regarding the mechanisms involved in conversion of sunlight energy into high-value products by photosynthetic microbes. The applied aspects of photosynthesis, current status of the problem, remaining challenges, and perspectives are discussed. Attention is paid to the metabolic pathways involved in biosynthesis of hydrogen, lipids, astaxanthin, and other carotenoids in green algae with a focus on the regulatory mechanisms induced in response to suboptimal conditions to provide energy homeostasis and photoprotection (Fig. 1). We also introduce a review discussing the potential of carbon nanotubes to enhance photosynthetic performance and productivity of photosynthetic microorganisms.

